# Workplace health coaching for chronic disease management in a maritime industry workforce: a mixed-methods evaluation

**DOI:** 10.3389/fpubh.2026.1760520

**Published:** 2026-07-06

**Authors:** Dhiya Mahirah, Lynette Teo, Mary Su-Lynn Chew, Clement Zhong-Hao Ho, Sing Ai Lee, Jane Lim

**Affiliations:** 1 Centre for Population Health Research and Implementation, Regional Health System, Singapore Health Services Pte Ltd., Singapore, Singapore; 2 Bridgepoint Health, Singapore, Singapore; 3 Health Services Research, Changi General Hospital, Singapore, Singapore

**Keywords:** chronic disease, health coaching, health promotion, occupational health services, program evaluation, Singapore

## Abstract

**Background:**

Workplace health coaching offers a promising approach to support behavior change and manage chronic diseases among working adults. This study evaluates the acceptability and preliminary outcomes of a pilot digitally supported health coaching program implemented in a maritime industry workforce in Singapore.

**Methods:**

A pragmatic mixed-methods program evaluation was conducted to examine both clinical outcomes and participant experiences. Quantitative data were collected at baseline and 6 months from 32 participants and included body mass index (BMI), blood pressure, lipid profile, and glycated hemoglobin (HbA1c). Health-related quality of life was assessed using the SF-12. Open-ended survey responses explored participants’ motivation, engagement, and perceptions of program components. Quantitative data were analyzed using Wilcoxon signed-rank tests, while participants’ qualitative responses were analyzed thematically.

**Results:**

Participants had a median age of 55 years (IQR: 44–61), and 89.1% were male, with representation across port operations, engineering, and administrative roles. Statistically significant improvements were observed in BMI, systolic blood pressure, triglycerides, and HDL cholesterol. Mental health-related quality of life improved, while HbA1c showed a small but statistically significant increase. Participants highlighted the motivational support structure, personalized guidance and encouragement from health coaches as key facilitators of engagement. Barriers included time constraints, digital fatigue, and usability issues. Participants reported adopting healthier routines and increased self-awareness, but noted difficulties maintaining those changes after the program ended.

**Conclusion:**

Findings suggest the feasibility and potential value of workplace health coaching in supporting employee well-being and chronic disease self-management. The results underscore the role of personalized and relational coaching approaches, and the importance of user-friendly digital tools and sustained support to maintain behavior change in workplace settings.

## 1 Introduction

Chronic conditions such as hypertension, diabetes, and hyperlipidemia are major contributors to the global disease burden, especially among working-age adults (1, 2). Globally, non-communicable diseases account for approximately 75% of all deaths (3). In Singapore, chronic conditions such as hypertension and hyperlipidemia affect approximately one-third of adults, while diabetes affects about one in ten, with prevalence increasing with age but already evident among working-age groups (4). In today’s work environments, sedentary job demands, long or irregular working hours, and psychosocial stressors can exacerbate these conditions and hinder effective self-management (5–7). Beyond their health effects, these conditions also have significant socioeconomic impacts, including reduced workforce participation, early retirement, and lost productivity from absenteeism and presenteeism, contributing to broader economic and labor market impacts, such as reduced employment and income (8–10).

Although these conditions can be effectively managed through ongoing lifestyle changes—such as healthier eating, regular physical activity, medication adherence, and stress management—individuals often struggle to sustain these behaviors due to limited time, competing demands, and psychosocial stressors (11, 12). Traditional healthcare systems, which tend to be episodic and treatment-focused, may not be well-equipped to provide the continuous behavioral support needed to help working adults integrate self-management practices into their daily routines (13, 14).

Health coaching has become a promising way to provide structured, person-centered support through trained coaches who apply behavior change theories to help individuals set goals, track progress, and sustain motivation (15). Evidence shows that health coaching can enhance clinical outcomes and health behaviors across different populations, especially those with chronic conditions (16, 17). While an increasing body of research supports its effectiveness in clinical and public health settings, workplace-based health coaching offers unique advantages for improving health behaviors and reducing healthcare use by leveraging the accessibility and convenience of the workplace to embed wellness activities into daily routines (18, 19). These approaches align with organizational efforts to promote employee well-being, lower the risk of chronic diseases, and maintain a healthy, productive workforce (20, 21).

However, there remains limited evidence on how these programs can be adapted and expanded in different organizational settings, especially regarding long-term engagement and understanding practical factors that influence participation. Studies evaluating workplace health coaching have mainly focused on biomedical outcomes and program adherence, with less attention to participants’ personal experiences, perceived value, and the contextual factors influencing engagement (22, 23). Furthermore, little is known about how workers experience the digital components of coaching programs, including usability, fatigue, and the adequacy of support after the initial engagement (24, 25). These gaps highlight the need for participant-informed evaluations to guide refinement of program design and digital engagement strategies.

This paper presents a pragmatic program evaluation of a pilot workplace health coaching program implemented in Singapore. The evaluation combined quantitative clinical data and quality-of-life measures with participant-reported feedback to examine both measurable outcome patterns and experiential aspects of engagement within a real-world occupational setting. While quantitative indicators provide information on changes in health outcomes, participant feedback offers additional insight into how individuals experienced the program and the contextual factors influencing engagement with coaching and digital components. Specifically, the evaluation aimed to: (1) examine preliminary changes in health-related and quality-of-life outcomes; (2) describe participants’ perceptions of the program’s acceptability and supportiveness; (3) identify facilitators and barriers to engagement during the coaching process; and (4) gather insights to inform recommendations for improving digital engagement and future workplace health promotion efforts.

## 2 Methods

### 2.1 Study design

This study provides a pragmatic evaluation of a pilot workplace-based health coaching program using administrative program data and participant feedback. The evaluation was conducted retrospectively following completion of the pilot and therefore reflects the data available from program monitoring rather than a prospectively designed mixed-methods study. Quantitative and qualitative data were gathered simultaneously and analyzed separately, with integration occurring at the interpretation stage. Rather than a predefined mixed methods design, this pragmatic QUANT + qual (20) approach combined measurable clinical and quality-of-life outcomes with participant feedback to provide a more comprehensive understanding of program acceptability, engagement processes, and patterns in preliminary outcome patterns.

### 2.2 Program description

The 6-month health coaching Ripple program aimed to support working adults with chronic conditions (e.g., hypertension, hyperlipidemia, type 2 diabetes) in adopting sustainable health behaviors. The program was implemented by Bridgepoint Health in collaboration with Northeast Medical Group (26) and involved a multidisciplinary team including a health coach, care coordinator, and medical doctor, providing coordinated, whole-person support for chronic disease management in the workplace (27).

The program combined monthly coaching sessions—conducted either in person or online—with regular digital follow-up. Coaching sessions were approximately 1 h in duration. On average, active participants attended one coaching session and had 11.6 interactions (such as chat messages, emails, or calls) with their health coach each month to support ongoing engagement between sessions. These interactions facilitated follow-up, feedback, and reinforcement of behavior change goals. In addition to communication, the digital platform enabled participants to input and monitor clinical indicators (e.g., weight, blood pressure, HbA1c, and lipid levels), track habit completion, and complete questionnaires throughout the program period. These data were accessible to coaches to inform and tailor subsequent coaching sessions. The platform also supported the sharing of contextual information, such as meal photos via in-app messaging, to facilitate personalized feedback and behavioral guidance.

Coaches were trained in motivational interviewing and used the Ripple program’s proprietary Habit Change Engine as a structured framework to guide coaching interactions and support behavior change across five domains: nutrition, physical activity, sleep, emotional well-being, and medication adherence. The approach was informed by established behavior change theories, including the Health Belief Model, Transtheoretical Model of Change, Social Cognitive Theory, and Self-Determination Theory, and was customized to meet participants’ cultural, linguistic, and work-related needs (28–31).

The employer supported implementation by subsidizing participation costs and coordinating on-site scheduling. Introductory coaching sessions were conducted during working hours in coordination with supervisors, while subsequent sessions were delivered on-site or online, with designated spaces provided by the employer to facilitate participation.

### 2.3 Participants and setting

Participants were employees from a private-sector workplace in Singapore who enrolled in the program between April and May 2023. The workforce represented a maritime industry setting, comprising employees working across port operations, engineering, and administrative functions. Eligible participants were working adults aged 21 years and older with one or more chronic conditions, such as hypertension, hyperlipidemia, or type 2 diabetes. Although the program primarily targeted employees with at least one self-reported chronic condition, participation was open to all interested employees, including those who did not report an existing diagnosis. Participation in the program was voluntary and employees self-enrolled after the program was introduced within the organization through internal workplace communications, resulting in a convenience sample of employees who chose to participate.

Participants’ job titles were classified according to the Singapore Standard Occupational Classification (32) and grouped into two main occupational groups: (1) technical and operational roles (e.g., engineers, technicians, and field or equipment specialists); and (2) professional and managerial roles (e.g., administrative, managerial, or executive positions).

### 2.4 Data collection

#### 2.4.1 Clinical and quality-of-life measures

Clinical and quality-of-life data were collected at baseline and at 6-month follow-up. Baseline measurements were taken during the first coaching sessions. Primary clinical outcomes included weight and body mass index (BMI), glycated hemoglobin (HbA1c), systolic and diastolic blood pressure, cholesterol, and triglyceride levels. Clinic-based indicators (e.g., HbA1c, lipid panel) were obtained from participants’ recent laboratory results conducted within approximately 1 month of the baseline or 6-month follow-up visit. If recent results were unavailable, participants were scheduled for testing at affiliated primary care clinics. Blood pressure was measured by coaches during sessions, while weight was self-reported. The same process was repeated at the follow-up data collection, with coaches prompted via the clinic management system to initiate collection. Missing data resulted from participant time constraints, refusal, physician advice that testing was unnecessary, or participant dropout from the program.

Body mass index (BMI) was categorized into overweight/obese categories (no: BMI < 23 kg/m^2^, yes: BMI ≥ 23 kg/m^2^) based on the WHO Asian BMI cutoff (33).

Health-related quality of life was measured using the 12-Item Short Form Health Survey (SF-12), a validated instrument that provides the Physical Component Summary (PCS) and Mental Component Summary (MCS) scores (34). Scores range from 0 to 100, with higher scores indicating better physical and mental health. The PCS reflects self-perceived physical functioning, pain, overall health, and role limitations caused by physical health, while the MCS assesses mental health, social functioning, vitality, and role limitations due to mental health (34).

#### 2.4.2 Participant feedback

Post-program survey responses were collected to gather participants’ perception of the program. The survey included Likert-scale items assessing overall experience, perceived behavioral impact, and digital engagement readiness. Several questions also included optional open-ended “Notes” fields that allowed participants to elaborate on their responses and provide additional feedback regarding program experiences, perceived benefits and suggestions for improvement. These open-ended survey responses formed the qualitative dataset analyzed in this study.

### 2.5 Ethics statement

This project was reviewed and determined to be exempt from full ethical review by the SingHealth Centralized Institutional Review Board (CIRB) because it involved the analysis of de-identified program data. Since no identifiable or sensitive personal information was used, formal CIRB approval was not required.

### 2.6 Data analysis

#### 2.6.1 Quantitative analysis

Descriptive analyses were performed to summarize participants’ characteristics and baseline health indicators. Categorical variables are reported as counts and percentages, while continuous variables are presented using medians and interquartile ranges (IQR). Given the small sample size and non-normal distribution of change scores, we used the Wilcoxon signed-rank test for all paired comparisons (35). Analyses were conducted using available paired baseline and follow-up data for each outcome. We report the median difference, IQR, *p*-value, and effect size for each outcome. Exploratory subgroup analyses were also carried out using Mann–Whitney U tests to compare 6-month change scores across gender, age groups, and occupational categories. All analyses were conducted using R (version 4.0).

#### 2.6.2 Qualitative analysis

Open-ended survey responses were analyzed using a descriptive thematic approach. Given the brief and evaluative nature of the written responses, the analysis focused on identifying recurrent patterns in participant feedback rather than generating theory or achieving thematic saturation. Initial deductive codes were informed by the evaluation objectives and survey prompts, while inductive coding allowed additional insights from participant reflections (36).

Two team members (MC and CH) independently reviewed and coded the responses to enhance analytic credibility. Coding differences were discussed and resolved through consensus with a third team member (DM). Given the relatively small corpus and consensus-based coding process, formal inter-rater reliability statistics were not calculated. Themes were iteratively reviewed and refined to reflect patterns in participant feedback and were supported with illustrative quotations. NVivo (Release 1.7.1) was used to manage and organize the data.

#### 2.6.3 Integration of findings

Quantitative and qualitative data were analyzed separately and then integrated during the interpretation phase using a merging approach (37). Quantitative results showed measurable changes in health and quality-of-life outcomes, while participant feedback offered contextual explanations and insights into facilitators, barriers, and behavioral processes that influenced engagement and observed trends. Integrating these findings allowed the qualitative responses to help explain patterns observed in the quantitative outcomes, providing a more comprehensive understanding of program acceptability and early outcome trends.

## 3 Results

### 3.1 Participant characteristics

A total of 55 employees completed the first coaching session and formed the baseline cohort for this evaluation, of whom 35 (63.6%) completed the 6-month follow-up. Program completion was defined as maintaining active engagement with a coach for approximately 6 months, meaning attending at least one coaching session or interaction each month. A participant flow diagram illustrating enrolment, program engagement, and completion is provided in Supplementary Figure S1.

Table 1 summarizes the baseline characteristics of all participants and those who completed 6 months of the program. The median age of the overall sample was 55 years (IQR: 44–61), and most participants were male (89.1%). Approximately two-thirds (67.3%) held technical or operational roles, while one-third (32.7%) occupied professional or managerial positions. The completion rate was higher among professional and managerial staff (83.3%) compared to those in technical or operational roles (54.1%). All six female participants completed the program, whereas 59.2% of male participants did. Completion rates were generally similar across age groups and comorbidity burden categories, though slightly lower among younger participants and those with three chronic conditions.

**Table 1:** Baseline characteristics of participants.

Characteristics	Completed 1st coaching session *n* = 55		Completed 6 months *n* = 35	
	*n*	%	*n*	%
Age (median, IQR)	55	44–61	56	45–61
<40	11	20.0	5	14.3
40–59	29	52.7	20	57.1
60 and above	15	27.3	10	28.6
Gender				
Male	49	89.1	29	82.9
Female	6	10.9	6	17.1
Ethnicity				
Chinese	34	61.8	26	74.3
Malay	13	23.6	5	14.3
Indian	7	12.7	4	11.4
Others	1	1.8	0	0.0
BMI, kg/m^2^ (median, IQR)	26.5	24.3–29.0	26.6	24.3–28.5
Normal (18.5–22.9)	5	9.3	3	8.6
Overweight (23.0–27.4)	30	55.6	21	60.0
Obese (≥27.5)	19	35.2	11	31.4
^*^Presence of chronic condition				
Type 2 Diabetes	16	29.1	10	28.6
Hyperlipidemia	31	56.4	20	57.1
Hypertension	29	52.7	16	45.7
*^*Comorbidity burden				
All three conditions	7	12.7	3	8.6
Two conditions	11	20.0	8	22.9
One condition	33	60.0	21	60.0
Occupational classification				
Technical and Operational roles	37	67.3	20	57.1
Professional and Managerial roles	18	32.7	15	42.9

^*^Participants could report more than one chronic condition. ^Categories are mutually exclusive; although eligibility criteria required participants to have ≥1 chronic condition, 4 participants did not self-report any of the listed conditions.

### 3.2 Changes in clinical and quality-of-life outcomes

Among participants with available paired data (approximately 28–35 across indicators), several clinical measures showed statistically significant changes over 6 months (see Table 2). Systolic blood pressure decreased significantly (*p* = 0.001), while diastolic blood pressure showed a significant increase (*p* = 0.005). BMI also showed significant reductions (*p* = 0.003). Changes were observed in lipid profiles: triglyceride levels decreased (*p* = 0.009), and HDL cholesterol increased (*p* = 0.040). HbA1c levels increased slightly over the six-month period (*p* = 0.043). For health-related quality of life, only the MCS significantly improved (*p* = 0.020).

**Table 2:** Clinical and quality of life outcomes at baseline and 6 months.

Outcome	*N*	Baseline: Median (IQR)	6 months: Median (IQR)	Median difference (IQR)	*p*-value	Effect size
Physical Component Score	27	50.0 (47.4–52.6)	52.5 (48.5–54.6)	2.7 (−3.2–5.1)	0.195	0.250
Mental Component Score	27	54.0 (48.0–59.4)	57.4 (55.2–59.5)	2.8 (−2.3–6.0)	0.020^*^	0.448
BMI (kg/m^2^)	34	26.7 (24.3–28.6)	25.9 (23.7–28.7)	−0.3 (−0.9–0.3)	0.005^**^	0.496
HbA1c (%)	35	5.7 (5.45–6.5)	5.8 (5.6–6.4)	0.1 (−0.1–0.2)	0.042^*^	0.392
Systolic BP (mmHg)	34	132.0 (120.3–140.0)	122.5 (118.0–130.0)	−6.0 (−15.0–−1.3)	0.001^**^	0.556
Diastolic BP (mmHg)	34	80.5 (75.0–87.3)	86.5 (77.0–92.8)	4.5 (1.0–12.0)	0.010^*^	0.443
Triglycerides (mmol/L)	33	1.8 (1.1–2.7)	1.5 (1.0–2.1)	−0.2 (−0.6–0.7)	0.009^**^	0.456
HDL (mmol/L)	33	1.2 (1.1–1.6)	1.3 (1.1–1.6)	0.1 (−0.0–0.1)	0.039^*^	0.372
LDL (mmol/L)	33	3.1 (2.4–3.9)	3.3 (2.6–4.0)	0.1 (−0.1–0.4)	0.082	0.307
Total Cholesterol (mmol/L)	33	5.3 (4.5–6.0)	5.7 (4.6–6.0)	0.1 (−0.2–0.4)	0.355	0.165

^*^Denotes significance: ^*^for *p* < 0.05, ^**^for *p* < 0.01.

Exploratory subgroup analyses by gender, age, and job type showed broadly similar patterns across subgroups (Supplementary Table S1). Given the small sample size and limited subgroup counts, these analyses should be interpreted as exploratory. The only statistically significant difference was a greater improvement in mental well-being (MCS) among male participants (*p* = 0.02), although this may reflect a small female sample size (*n* = 4).

To supplement the quantitative results, participants also completed a post-program survey assessing their overall satisfaction, perceived behavior change, and digital engagement preferences (Table 3). In addition to the structured survey ratings, participants were able to provide open-ended comments elaborating on their experiences with the program. Of the 35 participants who completed the program, 32 provided at least one written comment across the open-ended survey fields, generating 99 brief responses for qualitative analysis. These responses consisted of short reflections accompanying survey questions and provided additional context on participants’ experiences, perceived benefits, and suggestions for improvement. Qualitative analysis of these responses identified recurring patterns related to program engagement, perceived effectiveness of coaching support, lifestyle changes, and barriers to participation. An overview of the coding framework and representative quotations is provided in Supplementary Table S3.

**Table 3:** Participant feedback ratings (*n* = 32).

No.	Feedback item	% Positive response
1	Overall experience	90.6%
2	Helped make positive lifestyle change	84.4%
3	Motivation to make positive change	81.3%
4	Ability to create healthy habits	81.3%
5	Interested in using new Ripple™ App	78.1%
6	Would use mobile app to support lifestyle	81.3%
7	Currently use mobile app for routines	56.3%
8	Have access to information/resources	81.3%
9	Importance of access to health experts via app	65.6%

% Positive response indicates ≥4 on a 5-point scale or “Yes” on a Yes/No question. The full list of survey questions is available in the Supplementary Table S2.

### 3.3 Facilitators to engagement and perceived effectiveness of support

Survey results indicated that most participants reported positive perceptions of the program and its support features. In the post-program survey, 91% rated their overall experience positively, and 84% reported that the program helped them make positive lifestyle changes (Table 3). Similarly, 81% reported increased motivation and ability to maintain healthy habits, while two-thirds (66%) considered continued access to health experts through digital platforms important or very important.

Participants’ written comments provided further insights into these positive perceptions, particularly highlighting the role of personalized coaching support and ongoing encouragement in sustaining engagement with lifestyle changes. These self-reported positive perceptions are consistent with the observed changes in the Mental Component Score (MCS) of the SF-12, over the course of the program. Participants frequently attributed their motivation and progress to the encouragement and accountability provided by their health coaches, highlighting the role of motivational support and habit-building processes commonly emphasized in health coaching approaches.

*“Whatever in the past I wanted to do, i.e., 10 k steps to walk, now I have the motivation to start this habit.”; “My coach will constantly monitor and encourage me to go a bit more as I progress.”* (P10)

*“Coach has been guiding me in creating habits that works for me and my confidence level.”* (P29)

These reflections highlight the perceived importance of motivational structure and coaching support in maintaining engagement throughout the program.

### 3.4 Lifestyle and behavioral changes

Participants also described a range of lifestyle and behavioral changes that accompanied the observed changes in clinical outcomes. Several participants reported healthier eating habits, increased physical activity, and greater self-awareness in managing their health. Participants frequently attributed their progress to elements of the program such as goal-setting, progress monitoring, and regular feedback from coaches, which they perceived as fostering accountability and supporting habit formation.

*“I know my goal is to reduce my weight, but don't know where I go wrong. Coach is there to help assist and point in right direction, e.g., what is good to consume and what is bad for our health to consume.”* (P03)

*“I'm eating better and paying more attention to how and what I eat. Also, I've finally managed to stick to a regular exercise routine.”* (P27)

These accounts provide contextual insights alongside the observed changes in BMI, blood pressure, and lipid profiles over the 6-month period. Overall, participants’ feedback reflected increased self-management and awareness of health behaviors, with accountability through coaching and regular follow-ups identified as commonly cited factors associated with engagement in this program context.

### 3.5 Challenges to participation

While many participants reported positive experiences with the program, some also highlighted several challenges that affected engagement and the sustainability of lifestyle changes over time. Time constraints and competing priorities were often cited as reasons for decreased engagement. Some also reported challenges with the program’s digital components, including unfamiliarity with mobile apps, digital fatigue, and concerns about data security.

*“Reading materials on apps may not be universally useful, and logging into apps is not a habitual practice for me, which could affect my regular use of Ripple apps.”* (P02)

*“I don't use mobile apps regularly due to concerns about online scams, and I'm not particularly tech-savvy.”* (P22)

While most respondents (81%) expressed willingness to use a mobile health app, some participants expressed a preference for guidance from health professionals rather than relying primarily on digital tools. Participants also mentioned usability issues that impacted their experience, such as login difficulties and limited ability to edit habit logs.

*“Improvements are needed in the Ripple app's login re-authentication process. Additionally, the habit log feature could be more user-friendly, enabling clients to have greater control and edit their logs.”* (P19)

Collectively, these reflections highlight practical and technological challenges that may affect program adherence and point to opportunities for improving user experience and sustaining engagement over time.

## 4 Discussion

This pilot evaluation of a workplace health coaching program in Singapore suggested promising improvements in several clinical and quality-of-life outcomes over 6 months. As an exploratory evaluation conducted within a real-world workplace setting, the findings should be interpreted as preliminary indications of program impact rather than definitive evidence of effectiveness. We observed significant reductions in BMI and triglycerides, along with modest improvements in HDL cholesterol and mental health-related quality of life. Although systolic blood pressure improved significantly, diastolic blood pressure increased slightly over the study period. The reasons for this pattern are unclear and may reflect measurement variability or differences in participant characteristics; therefore, this finding should be interpreted cautiously. HbA1c also showed a small increase, while no notable changes occurred in physical health-related quality of life.

Qualitative data offered further insights into contextual factors and perceived mechanisms that may have contributed to the observed results. Participants emphasized the importance of motivational coaching, personalized support, and structured goal-setting in maintaining engagement and promoting behavior change. Several barriers were also identified, including time constraints, digital fatigue, and technical issues with the app. These findings suggest that the observed clinical improvements may be associated with coach-led behavioral modifications, increased self-monitoring, and enhanced health awareness reported by participants.

The observed improvements in BMI and lipid profiles align with participants’ descriptions of adopting healthier routines, including better diet and increased physical activity. These changes were accompanied by participants’ descriptions of consistent engagement with health coaches, which they perceived as strengthening motivation, accountability, and structured goal-setting (38, 39). Participant feedback emphasized how personalized coaching helped them turn intentions into lasting routines, indicating that the program’s behavioral framework, based on motivational interviewing and established behavior change theories, may have contributed to the improvements (40). These findings are consistent with evidence from health coaching and workplace-based interventions, which have demonstrated improvements in behavior change, weight, and cardiometabolic risk factors among individuals with chronic conditions and employee populations (41–43).

Beyond physical health changes, the observed improvement in mental health-related quality of life (MCS) may also reflect the psychosocial benefits of coaching. Participants described feeling more supported in making health changes, citing encouragement, goal reinforcement, better stress management, and regular follow-ups from coaches as key drivers of change. For working adults who often face job-related pressures and competing demands, such relational support may help buffer psychological strain and improve overall well-being (44). This structured emotional and relational support may have enhanced participants’ sense of control and psychological well-being, aligning with prior studies linking health coaching to reduced stress and better mental well-being, as well as workplace-based coaching interventions demonstrating similar psychological and behavioral benefits among employees (17, 23, 45, 46).

While several clinical indicators improved, a small but statistically significant increase in HbA1c levels was observed. As information on medication adjustments, diagnostic status, or other clinical factors was not available, the reasons for this change cannot be determined within the scope of this evaluation. Previous research has shown that short-term behavioral interventions may produce variable effects on glycemic outcomes, particularly when intervention duration is limited or intervention components differ across programs (47, 48). These findings highlight the importance of longer follow-up when evaluating glycemic outcomes in lifestyle interventions. A meta-analysis of health coaching interventions among individuals with type 2 diabetes found small but significant reductions in HbA1c, while noting that inconsistent intervention components, theoretical foundations, and delivery methods may contribute to mixed results (49). These findings suggest that both the intervention duration and program design may influence glycemic outcomes in coaching-based interventions. This variability is also reflected in workplace-based interventions, where heterogeneous program designs and levels of engagement contribute to mixed cardiometabolic outcomes (43).

While objective clinical markers generally improve, changes in the physical component of health-related quality of life (PCS) were less pronounced. This may reflect a disconnect between objective health improvements and participants’ subjective perception of physical function. Prior studies have shown that self-rated health is only moderately linked to objective clinical indicators and is heavily influenced by a combination of chronic disease status, functional limitations, and psychosocial stressors (50, 51). Since most of our participants had at least one chronic condition, it is plausible that their baseline perception of physical health was anchored by chronic morbidity, limiting their perceived improvement in physical functioning despite measurable progress (50, 51).

### 4.1 Implications for program improvement

To strengthen engagement at the individual level, program design should prioritize interpersonal elements that participants found most valuable. Participants often highlighted health coaches as the most important part of the program, especially for their motivational support, personalized guidance, and consistent follow-up. Maintaining this relational aspect through human support may be essential for sustaining engagement and encouraging behavior change (52, 53). Consideration should be given to retaining a hybrid model that combines in-person and digital coaching, particularly for participants who prefer face-to-face interaction (54).

Enhancing digital tools and delivery methods can boost usability and user engagement. Several suggestions have been made on how to improve the digital platform. Participants expressed a desire for more intuitive app features, better integration with existing devices such as smartwatches, Fitbit, or Healthy 365, and fewer barriers to access, like a simpler login process and clearer habit-tracking functions. These usability and interoperability issues are crucial for maintaining engagement with mobile health applications (55). Participants also recommended improvements in content and delivery, including weekly planning reminders and exercise videos to support goal-setting and habit formation. Providing timely, context-sensitive prompts that match an individual’s changing needs may be especially important for keeping users engaged and supporting habit formation (56). Participants appreciated when coaches adapted to their availability and routines. Offering flexible scheduling and alternative communication methods, such as platforms more familiar to users, could encourage wider participation, especially for those with time constraints or irregular work hours (57).

Although organizational-level factors were not systematically examined in this evaluation, employer support likely played an important role in facilitating participation. In this pilot program, the employer subsidized program costs and laboratory tests, coordinated introductory coaching sessions during working hours, and provided dedicated on-site spaces for coaching. Such structural support may reduce practical barriers to participation and enable employees to engage with health programs despite competing work demands. Future workplace health initiatives could benefit from more explicitly integrating health coaching within organizational structures, such as through protected participation time, supervisor support, and alignment with broader workplace wellness policies. Prior research indicates that workplace health programs are viewed as more valuable and sustainable when they are embedded within supportive organizational environments that reduce participation barriers and reinforce health-promoting behaviors (22, 58). Given that participants in this study frequently reported time constraints and competing responsibilities, addressing these structural factors may further enhance engagement and long-term program effectiveness (59).

### 4.2 Limitations and future research

Although this evaluation incorporated both quantitative and qualitative data, it was conducted as a pragmatic program evaluation rather than a prospectively designed mixed-methods study with predefined integration procedures. The qualitative component consisted of brief open-ended survey responses collected as part of program feedback rather than in-depth interviews or focus groups. Consequently, the qualitative data were intended to provide descriptive insights that complement quantitative findings rather than to achieve thematic saturation or theory generation. In addition, some clinical indicators (e.g., weight and blood pressure) were collected as part of routine program monitoring rather than a standardized research protocol, which may introduce measurement variability. Socio-demographic variables such as education level and other indicators of socio-economic status were not systematically collected and may have influenced participants’ engagement and responsiveness to the program.

The small sample size and the use of brief open-ended survey responses also limit the generalizability and depth of the findings. Participation in the program was voluntary, and employees who chose to enroll may have been more motivated to improve their health behaviors than those who did not participate, potentially introducing self-selection bias. Self-reported outcomes and participant feedback may also be subject to social desirability bias, which should be considered when interpreting the findings. In addition, participants were recruited from a single workplace with a workforce composition dominated by technical and operational roles, which contributed to the predominantly male sample observed in this evaluation. This gender imbalance may limit the generalizability of the findings to more gender-balanced workplace populations. Participant attrition and incomplete follow-up measurements further reduced statistical power. Although baseline characteristics of both enrolled and completing participants are reported, individuals who discontinued the program may differ systematically from those who remained engaged, which could introduce attrition bias when interpreting the observed outcome changes. Future studies incorporating in-depth interviews with both participants who dropped out and those who remained in the program could provide deeper insights into engagement mechanisms and barriers specific to working adults (60, 61).

In addition, this evaluation focused on participant experiences and outcomes and did not include perspectives from health coaches or organizational stakeholders, nor did it systematically examine workplace contextual factors that may influence program implementation and uptake. Incorporating these perspectives and applying implementation science frameworks could help clarify how workplace culture, facilitation processes, and organizational readiness influence program delivery and uptake (62). Evaluating implementation fidelity and understanding variations in delivery may also explain differences in participant experiences and the perceived value of the program (63). Additionally, the lack of long-term follow-up limits understanding of whether the observed benefits are sustained over time. Future research should incorporate longer follow-up periods and consider including objective behavioral metrics, such as physical activity tracking or dietary data, to better understand habit formation and maintenance within everyday work routines (64). Despite these limitations, the evaluation provides useful early insights into the feasibility and perceived value of workplace health coaching programs implemented in real-world organizational settings.

## 5 Conclusion

This evaluation suggests that workplace health coaching may contribute to short-term improvements in both clinical outcomes and self-reported well-being among employees managing chronic conditions. The combination of personalized coaching and regular supportive interactions that reinforce goals and motivation may be particularly valuable for working adults managing competing demands. Challenges with digital engagement emphasize the importance of designing tools that are intuitive and aligned with workers’ routines. These insights can inform improvements to health coaching delivery and guide the development of future workplace-based wellbeing programs. As organizations look for ways to maintain a healthy and productive workforce, findings from this pilot evaluation may help inform how employer-supported wellness initiatives are implemented and expanded in real-world occupational settings.

## Data Availability

The datasets presented in this article are not readily available because the participants did not provide written consent for their data to be shared externally. Requests to access the datasets should be directed to shq_dymm@nus.edu.sg.
