# Supplementary material for: Workplace health coaching for chronic disease management in a maritime industry workforce: a mixed-methods evaluation
Source: Front Public Health. 2026 Jul 6;14:1760520. doi: 10.3389/fpubh.2026.1760520 (PMC13381834; doi:10.3389/fpubh.2026.1760520)
Supplement: Supplementary file 1 [file Supplementary_File_1.docx]

**Figure S1: Participant flow through the workplace health coaching program.**

| Did not return complete 6-months measurements  n=20  Did not proceed with program  n=18  Provided follow up data  n=35  Registered for program  n=73  Participants engaged in program over 6 months  n=55  Completed first coaching session  n=55 |
| --- |

# Table S1: Six-month change in outcomes by participant subgroups

| **Variable** | **Gender (F vs M) – Median (IQR) Δ** | **p-value** | **Age (< 60 vs ≥ 60) – Median (IQR) Δ** | **p-value** | **Job Type (P/M vs T/O) – Median (IQR) Δ** | **p-value** |
| --- | --- | --- | --- | --- | --- | --- |
| **PCS** | –0.11 (–5.33–5.73) vs 2.65 (–3.02–5.10) | 0.682 | 2.78 (–3.33–5.29) vs 1.92 (–1.84–2.57) | 0.351 | 2.48 (–3.43–4.88) vs 2.67 (–3.02–5.10) | 0.845 |
| **MCS** | –3.37 (–4.96– –1.33) vs 4.97 (–0.65–7.21) | **0.020*** | 3.56 (–2.02–7.14) vs 1.67 (–2.02–5.06) | 0.816 | 2.54 (–2.47–7.56) vs 3.56 (–1.89–6.34) | 0.922 |
| **BMI (kg/m²)** | –0.34 (–0.78–0.15) vs –0.33 (–0.97–0.22) | 0.821 | –0.36 (–0.90–0.30) vs –0.32 (–0.99– –0.17) | 0.385 | –0.28 (–0.71–0.31) vs –0.42 (–1.15–0.06) | 0.323 |
| **HbA1c (%)** | 0.00 (0.00–0.08) vs 0.10 (–0.10–0.20) | 0.825 | 0.10 (0.00–0.20) vs 0.05 (–0.07–0.20) | 0.956 | 0.00 (–0.10–0.10) vs 0.10 (0.00–0.20) | 0.347 |
| **Systolic BP (mmHg)** | –6.00 (–12.50– –4.00) vs –6.00 (–18.25–0.25) | 0.910 | –8.00 (–18.25– –3.00) vs –2.50 (–8.50– –1.25) | 0.198 | –4.00 (–11.50– –0.50) vs –8.00 (–21.50– –2.00) | 0.476 |
| **Diastolic BP (mmHg)** | 2.50 (1.00–7.00) vs 5.00 (0.00–12.25) | 0.603 | 5.50 (1.00–14.00) vs 2.50 (–2.00–5.50) | 0.22 | 4.00 (–2.00–10.00) vs 5.00 (1.00–14.50) | 0.366 |
| **Triglycerides (mmol/L)** | –0.10 (–0.15–0.03) vs –0.18 (–0.74–0.06) | 0.283 | –0.11 (–0.53–0.09) vs –0.63 (–0.99– –0.12) | 0.166 | –0.14 (–0.53– –0.06) vs –0.17 (–0.64–0.10) | 0.813 |
| **HDL (mmol/L)** | –0.04 (–0.10–0.05) vs 0.06 (–0.02–0.15) | 0.243 | 0.03 (–0.03–0.12) vs 0.13 (0.05–0.18) | 0.185 | –0.01 (–0.03–0.12) vs 0.06 (–0.03–0.15) | 0.784 |
| **LDL (mmol/L)** | –0.04 (–0.13–0.01) vs 0.26 (–0.04–0.46) | 0.107 | 0.01 (–0.14–0.30) vs 0.41 (0.08–0.88) | 0.093 | 0.15 (–0.03–0.36) vs 0.06 (–0.20–0.53) | 0.927 |
| **Total Cholesterol (mmol/L)** | –0.06 (–0.18–0.08) vs 0.10 (–0.20–0.49) | 0.315 | 0.00 (–0.25–0.35) vs 0.31 (0.07–0.54) | 0.115 | 0.10 (–0.08–0.25) vs 0.03 (–0.33–0.50) | 0.757 |

*p<0.05 (Mann-Whitney U test).

# Table S2: Participant Survey questions and response options

| **No.** | **Feedback item** | **Survey Question** | **Response Options** |
| --- | --- | --- | --- |
| 1 | Overall experience | Overall, how would you rate your experience in the Ripple™ programme? | 1 (very dissatisfied) – 5 (very satisfied) |
| 2 | Helped make positive lifestyle change | How well has the Ripple™ programme helped you in making positive changes in your lifestyle and routines? | 1 (not at all) – 5 (extremely well) |
| 3 | Motivation to make positive change | Your motivation to make a positive healthy change in your lifestyle has … | 1 (decreased) – 5 (increased) |
| 4 | Ability to create healthy habits | Your ability to create positive healthy habits in your routines has … | 1 (decreased) – 5 (increased) |
| 5 | Interested in using new Ripple™ App | Would you be interested in using the new Ripple™ App with features that encourage consistent engagement for promoting a healthy lifestyle? | Yes / No |
| 6 | Would use mobile app to support lifestyle | Do you see yourself using a mobile app to support your healthy lifestyle? | Yes / No |
| 7 | Currently use mobile app for routines | Do you use any mobile app today to help you manage your daily routine? | Yes / No |
| 8 | Have access to information/resources | Do you have access to relevant information, documents or quick guides that help you make healthier choices in your day to day? | Yes / No |
| 9 | Importance of access to health experts via app | How important is it to have access to health experts through a digital app? | 1 (not important) – 5 (very important) |

**Table S3: Summary of Themes and Sub-themes from Qualitative Data**

| **Theme** | **Sub-theme** | **Illustrative Quote** |
| --- | --- | --- |
| **A: Facilitators to engagement and perceived effectiveness of support** | 1. Motivational programme design | “Whatever in the past I wanted to do i.e 10k steps to walk, now I have the motivation to start this habit.” |
|  | 1. Health coach support and encouragement | “My coach will constantly monitor and encourage me to go a bit more as I progress.” |
|  | 1. Personalisation of coaching | “Coach able to adjust to my busy timeslot” |
|  | 1. Value of expert support | “Coach has been guiding in creating habits that works for me and my confidence level.” |
|  | 1. Sustaining motivation and habit formation | “Life gets really busy with personal and work commitments. Having more reminders and encouragement from the program would be really helpful. Sending in a food log has been a good way to remind myself to stay healthy.” |
| **B:**  **Lifestyle & Behavioural Changes** | 1. Lifestyle change and self-management | “I've become more mindful of my eating habits and increased my physical activity.” |
|  | 1. Health improvements and outcomes | “Thanks to my coach, I really see improvements. Until my company doctor say don't need to see him, … I save up money.” |
| **C:**  **Challenges to Participation** | 1. Time and competing priorities | “At times, I find myself too busy at work, making it challenging to consistently maintain healthy habits.” |
|  | 1. Difficulty sustaining behaviour change | “This program has made me more health-conscious and helpful, yet I occasionally struggle with my personal motivation to make lifestyle changes.” |
|  | 1. Preference for human coaching over digital-only tools | “Beneficial to seek guidance from health experts rather than solely relying on AI, as their advice is easier to comprehend and more practical.” |
|  | 1. Digital fatigue and low app engagement | “Reading materials on apps may not be universally useful, and logging into apps is not a habitual practice for me, which could affect my regular use of Ripple apps.” |
|  | 1. Technical and trust-related concerns | “I don't use mobile apps regularly due to concerns about online scams, and I'm not particularly tech-savvy.” |
